# Non-Steroidal Anti-Inflammatory Drugs Do Not Influence the Urinary Testosterone/Epitestosterone Glucuronide Ratio

**DOI:** 10.3389/fendo.2013.00051

**Published:** 2013-05-16

**Authors:** Jonas Lundmark, Nina Gårevik, John-Olof Thörngren, Mats Garle, Lena Ekström, Anders Rane, Jenny J. Schulze

**Affiliations:** ^1^Division of Clinical Pharmacology, Department of Laboratory Medicine, Karolinska InstitutetStockholm, Sweden

**Keywords:** NSAID, testosterone, epitestosterone, diclofenac, ibuprofen, UGT2B17, T/E ratio

## Abstract

The UDP Glucuronosyl Transferase (UGT) enzymes are important in the pharmacokinetics, and conjugation, of a variety of drugs including non-steroidal anti-inflammatory drugs (NSAIDs) as well as anabolic androgenic steroids (AAS). Testosterone glucuronidation capacity is strongly associated with a deletion polymorphism in the UGT2B17 gene. As the use of high doses of NSAIDs has been observed in athletes there is a risk for a drug–drug interaction that may influence the doping tests for AAS. *In vitro* studies show inhibitory potential on UGT2B7, 2B15, and 2B17 enzymes by NSAIDs. The aim of this study was to investigate if concomitant use of NSAIDs and a single dose of testosterone enanthate would affect the excretion rate of testosterone and epitestosterone glucuronide (TG and EG) as well as the T/E ratio, thereby affecting the outcome of the testosterone doping test. The study was designed as an open, randomized, cross-over study with subjects being their own control. The 23 male healthy volunteers, with either two, one or no allele (ins/ins, ins/del, or del/del) of the UGT2B17 gene, received the maximum recommended dose of NSAID (Ibuprofen or Diclofenac) for 6 days. On day three, 500 mg of testosterone enanthate was administered. Spot urine samples were collected for 17 days. After a wash-out period of 4 months the volunteers received 500 mg testosterone enanthate only, with subsequent spot urine collection for 14 days. The glucuronides of testosterone and epitestosterone were quantified. NSAIDs did not affect the excretion of TG or EG before the administration of testosterone. The concomitant use of NSAIDs and testosterone slightly increased the TG excretion while the EG excretion was less suppressed compared to testosterone use only. The effects of the NSAIDs on the TG and EG excretion did not differ between the UGT2B17 genotype groups. In conclusion, the outcome of testosterone doping tests does not seem to be affected by the use of NSAIDs.

## Introduction

Testosterone is one of the most commonly abused anabolic androgenic steroids (AAS) within doping in sports and for enhancement of physical performance (Handelsman and Heather, 2008). Testosterone and other anabolic compounds are prohibited in sports by the World Anti-Doping Agency (WADA). In 2011 anabolic agents represented the most frequently reported adverse analytical findings and atypical findings (59.4%) by accredited doping laboratories. Among these, elevated testosterone/epitestosterone ratios represented 60.0% of the findings, although only 10% of these were adverse analytical findings (WADA, 2011).

To discriminate exogenous testosterone from testosterone of endogenous origin the urinary ratio of testosterone glucuronide (TG) to epitestosterone glucuronide (EG) (T/E ratio) is used (Donike et al., 1983, 1985). Based on population studies (Donike et al., 1983, 1985) a normal T/E ratio would be ∼1.0 and a T/E ratio above six was initially considered suspicious of doping. However, additional knowledge showed that Asian individuals excreted low amounts of TG, and as a result low T/E ratios increasing the risk of false negative doping test results (Park et al., 1990; de la Torre et al., 1997). Due to these findings the T/E ratio was lowered to 4.0 in 2004.

Testosterone is inactivated, and excreted in urine, mainly as glucuronide conjugates, the formation of which is catalyzed by UDP-glucuronosyltransferases (UGTs). These enzymes play a key role in the homeostasis of a number of endogenous molecules including steroid hormones (Belanger et al., 2003). The UGT super family is subdivided into UGT1A, UGT2A, and UGT2B families based on sequence identity (Mackenzie et al., 1997). In humans UGTs 2B7, 2B15, and 2B17 are the main catalysts of the glucuronidation of androgens and their metabolites (Belanger et al., 2003). Testosterone glucuronidation is mainly dependent on UGT2B17 and to a lesser extent UGT2B15 (Turgeon et al., 2001) whereas epitestosterone is conjugated by UGT2B7 (Coffman et al., 1998; Sten et al., 2009a).

The human UGT2B genes are clustered on chromosome 4q13-21.1 and encode seven functional enzymes: UGT2B4, B7, B10, B11, B15, B17, and B28 (Guillemette, 2003). *In vivo*, UGT2B17 has been identified as the main enzyme in testosterone glucuronidation where a gene deletion in *UGT2B17* was associated by us with low, or negligible, excretion of testosterone in urine (Jakobsson et al., 2006). All subjects devoid of UGT2B17 had a T/E ratio below 0.4. This polymorphism was considerably more common in a Korean Asian than in a Swedish Caucasian population, with 66.7 and 9.3% deletion/deletion (del/del) homozygotes, respectively (Jakobsson et al., 2006). This correlates to earlier findings of low T/E in Asians as described above. Further, after testosterone administration individuals devoid of the *UGT2B17* gene rarely exceed this cut-off ratio (Schulze et al., 2008b). Individuals carrying one (ins/del) or two (ins/ins) copies of this gene clearly passed the cut-off ratio and some even exceeded the cut-off level prior to challenge with testosterone.

The UGT enzymes are important in the pharmacokinetics, and conjugation, of a variety of drugs including non-steroidal anti-inflammatory drugs (NSAIDs) (Davies, 1998; Kuehl et al., 2005). NSAIDs are a class of therapeutic agents used in the treatment of pain and inflammation and are commonly used by athletes. In fact, according to recent studies, inappropriate use of high doses and concomitant use of several different NSAIDs has been observed in athletes (Alaranta et al., 2008; Warden, 2009). Since steroids and NSAIDs are both inactivated by UGT enzymes there is a risk for a drug–drug interaction (Kiang et al., 2005). *In vitro* studies show inhibitory potential on UGT2B7 (Mano et al., 2007), 2B15 and 2B17 enzymes by NSAIDs (Sten et al., 2009b). In the latter study both diclofenac and ibuprofen inhibited testosterone glucuronidation in liver microsomes, as well as recombinant UGT2B15 and UGT2B17 enzymes. However, epitestosterone glucuronidation activity in human liver microsomes was largely insensitive to ibuprofen and diclofenac. To our knowledge this has not been studied *in vivo*.

The aim of this study was to investigate in healthy male volunteers whether a concomitant use of NSAIDs (Ibuprofen or Diclofenac) and a single dose of testosterone enanthate would affect the excretion rate of TG and EG, and hence the T/E ratio, to the extent that it could affect the outcome of the testosterone doping test.

## Materials and Methods

### Subjects

The target group was healthy male volunteers aged 18–50 years. A total number of 33 subjects were genotyped for the *UGT2B17* deletion polymorphism to fill the pre-determined number of approximately 10 subjects in each of the three different genotype panels (ins/ins, ins/del and del/del). All subjects originated from different ethnicities and therefore the genotype frequencies in this sample are not representative of any particular population. In this particular genotyped population group 28% were homozygous for the gene deletion (del/del), 33% were heterozygous (ins/del), and 39% had two copies of the gene (ins/ins). In total, eight del/del, seven ins/del, and eight ins/ins participants completed the study. Study population characteristics are presented in Table 1. In addition to genotyping, all subjects underwent a medical examination including laboratory tests before enrollment to exclude the possibility of any disease. No pharmaceutical compounds other than those in this study were allowed. Further inclusion-criteria included a negative screening for illegal drugs, AAS, HIV, and hepatitis B or C virus. For inclusion it was also required that the subject was not a member of any organization belonging to the Swedish Sports Confederation, had been diagnosed and/or treated for a malignancy within the past 5 years or having a known allergy to the study substances. All participants gave informed consent consistent with the approval of the Ethics Review Board at Karolinska Institutet in Stockholm. Two subjects were excluded prior to the start of the study due to concomitant medication. The genotyped subjects that did not participate were dropouts (*n* = 8). The study (protocol number 2007-002655-16) was conducted according to the Helsinki declaration and the ICH Harmonized Tripartite Guideline for Good Clinical Practice.

**Table 1:** **Study population characteristics at screening**.

*UGT2B17* Genotype	Age (years)	Height (cm)	Weight (kg)	BMI (kg/m^2^)
del/del (*n* = 8)	29.2 ± 4.3	180 ± 6.9	80.6 ± 9.7	24.8 ± 2.7
ins/del (*n* = 7)	31.1 ± 4.7	179 ± 6.1	81.4 ± 9.2	25.4 ± 2.8
ins/ins (*n* = 8)	28.1 ± 5.1	181 ± 5.2	79.6 ± 6.1	24.3 ± 2.3

### Genotyping of *UGT2B17*

The copy number of *UGT2B17* was determined in the 23 males included in the study. The numbers of *UGT2B17* genes quantified with a real-time PCR analysis, using the expression of albumin as an endogenous control. The genotyping methods are based on the 5′-nuclease activity method (TaqMan^®^) employing two primers and two fluorescent labeled probes in a real-time based assay as earlier described (Schulze et al., 2008a).

### Design

**Figure 1:**
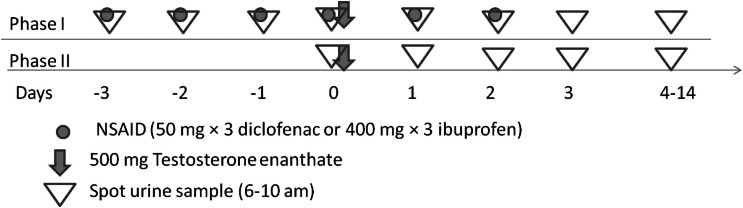
**Illustration of the open, randomized, cross-over study with subjects being their own control**. The wash-out period was at least 4 months.

The study was designed as an open, randomized, cross-over study with subjects being their own control (Figure 1). In the Phase I, starting 3 days before (−3) administration of Testoviron^®^-Depot, subjects received either diclofenac (Voltaren^®^, *n* = 13) 50 mg × 3 or ibuprofen (Ipren^®^, *n* = 10) 400 mg × 3 for six consecutive days (ending on day-2).

On day-0 all participants from the different genotype panels were given a single dose of 500 mg testosterone enanthate in ricinus oil as a single intramuscular dose of Testoviron^®^ – Depot (kindly provided by Bayer Schering Pharma) equivalent to 360 mg testosterone. Before administration of the testosterone, urine samples were collected during 4 days (days-3, -2, -1, and 0). Samples of urine were further collected for 14 days, all between 06–10 am. In the Phase II all participants were given a single dose of 500 mg testosterone enanthate in ricinus oil as a single intramuscular dose of Testoviron^®^-Depot equivalent to 360 mg testosterone. A baseline urine sample was collected before the injection and further for 14 days after the injection, all between 06:00 and 10:00 a.m. The wash-out period between the two phases was at least 4 months. Adverse drug reactions (ADRs) were monitored from inclusion of each phase until day-14 after administration of testosterone. No major ADRs were registered. No follow-up was needed.

### Blood and urine samples

Venous blood was obtained from the cubital vein and collected in EDTA tubes for DNA extraction. The urine samples were collected and kept refrigerated for maximum 48 h and then frozen at −20°C. All samples were collected between 06:00 and 10:00 a.m.

### Urine analyses

Urinary unconjugated steroids and steroid glucuronides were determined using liquid chromatography-mass spectrometry (LC/MS) as described previously (Schulze et al., 2011). The method was modified to only quantify the glucuronides of testosterone and epitestosterone. The day-to-day variation of the instrument was minimized using the mixture of authentic standards analyzed with every batch of samples.

### Data analyses

Integration, calibration, and data evaluation was performed by the TargetLynx software (Waters Associates, Manchester, UK).

The between-subject variation in urine dilution was corrected for by dividing the concentration values by the urinary creatinine (cr) concentration, which was determined by colorimetric analysis (DRI Creatinine-Detect Test; Thermo Fisher Scientific, Waltham, MA, USA). Statistical analyses for comparison of hormonal levels of TG, EG, and T/E were performed with Student’s two-tailed *t* test or Mann–Whitney *U* test according to distribution of the data. When comparing three groups; ANOVA with Tukey’s *post hoc* test or Kruskal–Wallis with Dunn’s *post hoc* test according to distribution. *p* < 0.05 regarded as significant.

## Results

### Baseline urinary steroids

The average baseline urinary glucuronidated testosterone (TG) and epitestosterone (EG) concentrations and T/E ratios for Phase I before and after 3 days of NSAID administration are presented in Table 2. Neither the TG levels, EG levels nor the T/E ratio were affected by NSAID administration. For the ins/del group the TG levels decreased slightly after 3 days of NSAID administration, however, this decrease was not statistically significant (*p* = 0.13). As expected, the TG levels and T/E ratio differed significantly between the del/del group and the other two genotype groups. There was no difference in the EG levels. No difference in baseline values between individuals aimed for receiving ibuprofen or diclofenac was observed.

**Table 2:** **Baseline urinary androgen glucuronide levels (A) before and after 3 days of NSAID administration Phase I and (B) in Phase II**.

**(A)**						

*UGT2B17* Genotype	Testosterone (ng/μmol cr)		Epitestosterone (ng/μmol cr)		T/E ratio	
	Day-3**	Day-0**	Day-3	Day-0	Day-3**	Day-0**
del/del (*n* = 8)	0.33 ± 0.34	0.38 ± 0.24	5.2 ± 2.4	5.2 ± 1.9	0.07 ± 0.06	0.08 ± 0.05
ins/del (*n* = 7)	6.1 ± 2.0	4.4 ± 1.9	6.3 ± 3.2	5.8 ± 4.0	1.2 ± 0.70	0.90 ± 0.39
ins/ins (*n* = 8)	5.5 ± 2.3	6.6 ± 3.0	3.3 ± 2.0	3.5 ± 1.5	2.0 ± 0.79	2.0 ± 0.65

**(B)**						

**UGT2B17 Genotype**	Testosterone G *(ng/μmol cr)	**Epitestosterone (ng/μmol cr)**	T/E ratio*			
del/del (*n* = 8)	0.46 ± 0.33	4.5 ± 3.9	0.15 ± 0.11			
ins/del (*n* = 7)	4.0 ± 1.1	5.0 ± 2.2	0.94 ± 0.60			
ins/ins (*n* = 8)	8.7 ± 5.6	4.2 ± 2.6	2.1 ± 0.77			

The values are given as the mean ± standard deviation. Asterisks denote a statistically significant difference (*p < 0.001; **p < 0.0001) between the genotype as determined by ANOVA.

The average baseline T/E ratios for Phase II are presented in Table 2. Similar to Phase I the TG levels and the T/E ratio differed significantly between the genotype groups.

### Urinary steroid profile after testosterone administration

**Figure 2:**
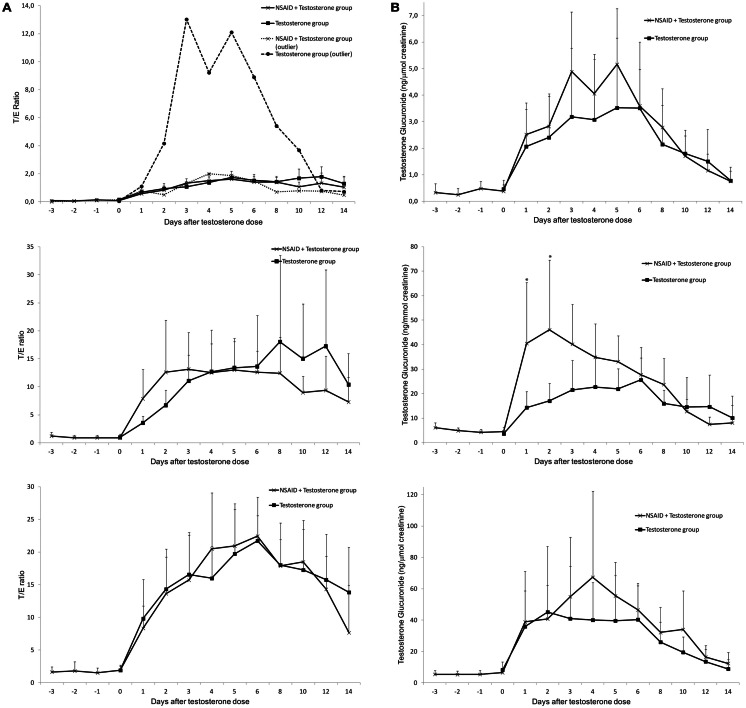
**Average urinary testosterone/epitestosterone ratios (A) and urinary testosterone glucuronide excretion (ng/μmol creatinine) (B) during 14 days in UGT2B17 del/del (top), ins/del (middle) and ins/ins (bottom) genotype groups of healthy volunteer males**. The subjects were followed during two administration cycles with at least 4 months wash-out between the cycles. During one cycle NSAID was administered for 6 days (day-3 to day-2). In both cycles an intramuscular dose of 500 mg testosterone enanthate was administered on day-0. Asterisks denote statistically significant differences between the groups (*p < 0.05; **p < 0.01). Vertical bars denote standard deviations.

The T/E ratios in Phase I (NSAID + T) and Phase II (T only) for the different genotype panels after testosterone dose are shown in Figure 2A. There were no statistically significant differences between Phase I and Phase II. In the del/del group there was one individual with EG levels decreasing much less in Phase I compared to Phase II, while his TG levels were similar in both Phase I and Phase II. This individual is shown as a dotted line in Figure 2A (top).

The TG increased slightly more in Phase I (NSAID + T) compared to Phase II (T only) after dose in all genotype groups (Figure 2B). However the increase was only significant in the ins/del genotype group on day-1 (*p* = 0.02) and day-2 (*p* = 0.05). Taken all genotype groups together the maximum increase in TG excretion compared to baseline was about 80% higher in Phase I (NSAID + T) compared to Phase II (T only). There was no statistically significant difference between the diclofenac group compared to the ibuprofen group.

**Figure 3:**
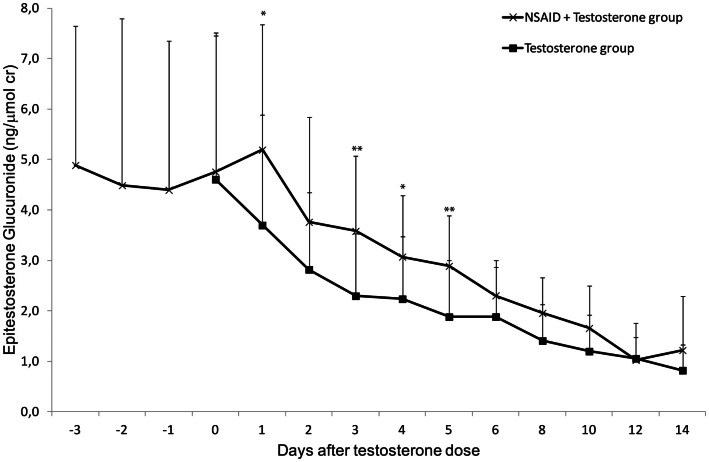
**Average urinary epitestosterone glucuronide excretion (ng/μmol creatinine) during 14 days in healthy volunteer males**. The subjects were followed during two administration cycles with at least 4 months wash-out between the cycles. During one cycle NSAID was administered for 6 days (day-3 to day-2). In both cycles an intramuscular dose of 500 mg testosterone enanthate was administered on day-0. Asterisks denote statistically significant differences between the groups (*p < 0.05; **p < 0.01) Vertical bars denote standard deviations.

The excretion of EG decreased steadily to low levels after the testosterone dose as expected. The decrease was, however, more pronounced in Phase II (T only) compared to Phase I (NSAID + T) in all genotype groups the first 5 days after the testosterone dose. This corresponds to the time the study subjects still had relatively high concentrations of NSAID in their circulation. As the EG excretion is not dependent on *UGT2B17* genotype the three *UGT2B17* genotype panels were grouped for increased statistical power (Figure 3). On days 1–5 after the testosterone dose, the EG concentrations were significantly less suppressed in Phase I (+NSAID) compared to Phase II (*p* = 0.04 − 0.003) with the exception of day-2 (*p* = 0.08). No difference was seen between the diclofenac and the ibuprofen group.

Taken together the results indicate that NSAID slightly induces TG and decreases suppression of EG excretion, hence there will be no effect on the T/E ratio.

## Discussion

In this study, for the first time, we aimed to study if NSAIDs affect testosterone glucuronidation, epitestosterone glucuronidation and hence T/E ratios *in vivo*. Based on previous *in vitro* data (Sten et al., 2009b) the hypothesis was that NSAIDs would interact with the glucuronidation of testosterone but not epitestosterone leading to lower TG excretion and lower T/E ratios. However, the opposite was seen, with a minor increase in TG and less suppression of EG excretion leading to unchanged T/E ratios.

In our *in vitro* study (Sten et al., 2009b) the IC_50_ values for the inhibition of testosterone glucuronidation by ibuprofen was 213 (95% CI; 129–354) μM and by diclofenac 64 (95% CI;53–80) in human liver microsomes (Sten et al., 2009b). Therapeutic plasma concentrations for ibuprofen are 73–146 μM, which are in the same range as the IC50 value. For diclofenac the therapeutic plasma concentrations are considerably lower (about 5 μM) than the *in vitro* IC50 value. We still decided to include diclofenac in the *in vivo* study, as this is a very common NSAID used by athletes.

It is not straight forward to predict inhibitory interactions involving glucuronidated drugs from *in vitro* data (Miners et al., 2006) and references therein. We have shown that testosterone glucuronidation rate *in vitro* in liver microsomes was only twofold higher in livers with one or two copies of the *UGT2B17* gene compared to *UGT2B17* del/del livers (Jakobsson et al., 2006). *In vivo*, urinary levels of TG in *UGT2B17* ins/ins and ins/del individuals are over 10 times higher than TG levels in *UGT2B17* del/del individuals (this study; Jakobsson et al., 2006; Schulze et al., 2008b). In addition, in our *in vitro* experiment only liver microsomes and recombinant enzymes were studied (Sten et al., 2009b), while the UGT2B enzymes are highly expressed in several different tissues (Nakamura et al., 2008).

Our intention with this study was aimed at mimicking a situation that doping laboratories may face when athletes are taking NSAIDs. We conclude that NSAIDs, even in large doses, will not have any significant effect on testosterone doping tests results, at least not with injected testosterone esters. Oral formulations of testosterone are metabolized in the liver to a much higher extent than injectable testosterone esters. It would be interesting to investigate if oral formulations of testosterone together with NSAID would show different results. It is possible that this setting would be more similar to our *in vitro* investigation where liver microsomes were used (Sten et al., 2009b).

To our knowledge the interaction of NSAID with the glucuronidation enzymes has not been extensively studied *in vivo*. Van der Logt et al. (2004) demonstrated increased UGT enzyme activities in rats treated with ibuprofen in intestine but not in liver.

The plasma concentrations of ibuprofen and diclofenac are known to be highly dependent on polymorphisms in CYP2C9 and CYP2C8 (Garcia-Martin et al., 2004; Zhou et al., 2010). Indeed, the TG excretion increased more than 20% in 16 study subjects, was unchanged in six and was decreased in one subject after the testosterone injection with concomitant use of NSAID compared to a testosterone injection only.

We chose to use a large dose of testosterone enanthate in order to have plasma concentrations as close as possible to our previous *in vitro* investigation (Sten et al., 2009b), and also, since our study group was quite small, to be able to detect any changes in TG and EG excretion. It would be interesting to use different formulations of testosterone rather than different types of NSAIDs.

Several *in vitro* studies have shown UGT2B inhibition with different types of food or drink (Jenkinson et al., 2012a,b) or drugs (Sten et al., 2009b) and speculated that this will affect the testosterone excretion and testosterone doping tests. Our *in vivo* study does not confirm previous *in vitro* results, rather the opposite, and this should be taken into consideration when extrapolating *in vitro* results to the *in vivo* situation.

In conclusion, the concomitant use of NSAIDs and testosterone esters slightly increases the TG excretion while the EG excretion is less suppressed compared to testosterone use only. The T/E ratios are not affected by NSAID intake either before or after the testosterone administration.

## Conflict of Interest Statement

The authors declare that the research was conducted in the absence of any commercial or financial relationships that could be construed as a potential conflict of interest.
